# Adequate life-expectancy reconstruction for adult human mortality data

**DOI:** 10.1371/journal.pone.0198485

**Published:** 2018-06-04

**Authors:** László Németh, Trifon I. Missov

**Affiliations:** 1 Laboratory of Survival and Longevity, Max Planck Institute for Demographic Research, Rostock, Germany; 2 Hungarian Demographic Research Institute, Budapest, Hungary; University of West London, UNITED KINGDOM

## Abstract

Mortality information of populations is aggregated in life tables that serve as a basis for calculation of life expectancy and various life disparity measures. Conventional life-table methods address right-censoring inadequately by assuming a constant hazard in the last open-ended age group. As a result, life expectancy can be substantially distorted, especially in the case when the last age group in a life table contains a large proportion of the population. Previous research suggests addressing censoring in a gamma-Gompertz-Makeham model setting as this framework incorporates all major features of adult mortality. In this article, we quantify the difference between gamma-Gompertz-Makeham life expectancy values and those published in the largest publicly available high-quality life-table databases for human populations, drawing attention to populations for which life expectancy values should be reconsidered. We also advocate the use of gamma-Gompertz-Makeham life expectancy for three reasons. First, model-based life-expectancy calculation successfully handles the problem of data quality or availability, resulting in severe censoring due to the unification of a substantial number of deaths in the last open-end age group. Second, model-based life expectancies are preferable in the case of data scarcity, i.e. when data contain numerous age groups with zero death counts: here, we provide an example of hunter-gatherer populations. Third, gamma-Gompertz-Makeham-based life expectancy values are almost identical to the ones provided by the major high-quality human mortality databases that use more complicated procedures. Applying a gamma-Gompertz-Makeham model to adult mortality data can be used to revise life-expectancy trends for historical populations that usually serve as input for mortality forecasts.

## Introduction

Mortality of populations is summarized in life tables. The latter contain certain measures of mortality (e.g. remaining life expectancy, survival probability at age *x*), but researchers also calculate other characteristics of the distribution of deaths based on life-table information (e.g. Gini coefficient, Human Development Index, etc.) to use them as input in public and health policy making, insurance and investments.

Life tables aggregate mortality information above a certain age, i.e., an open-ended age group “closes” the life table. For this age group, researchers assume different types of behavior for the risk of dying that affect directly remaining life expectancy values at each age. The most frequently used “closing procedure” is based on the constant-hazard assumption in the open-ended age group [1], which does not reflect the conventional treatment of right censoring in survival data. As a result, mortality measures can be distorted, especially when the open-ended age group contains a large proportion of the population [2]. This is often the case for countries with low-quality mortality data at later ages. The United Nations (UN) estimated life table of females in the world for the years 2010-2015 contains 32% of the population surviving until the last 85+ age group. For Bangladesh the figure is 24%, for Thailand almost 38%, for Vietnam almost 47%, for India more than 20%, and for Brazil more than 40% (see [3] for details). Even though these shares might be influenced by age misreporting, it is still important to address censoring adequately when calculating life expectancy for such populations.

Other widely used methods for “closing” a life table are based on either modifying the level of the constant hazard [4] or making other assumptions about the risk of dying in the open-ended age group. However, closing a life table by any of these methods can result in serious distortions of life expectancy values. Missov et al. [2] discuss the potential bias in life expectancy arising from applying these methods and recommend addressing right-censoring in a parametric model setting. Using a continuous (parametric) model has at least three major advantages: first, the parameters of the fitted model have meaningful demographic interpretation, second, it aids reconstructing age-specific quantities from abridged or grouped data, and third, it provides mortality measure estimates, e.g. life expectancy, at non-integer ages.

It has been known since Gompertz [5] that a large part of adult human mortality follows an exponentially increasing curve. Empirical data, though, show deviations at both ends of this log-linear pattern. On the one hand, mainly due to extrinsic factors, young adult mortality is higher at the left end of the log-linear curve. On the other hand, mortality rates tend to slow down at older ages. Therefore, it is desirable to find a parsimonious model that not only addresses censoring adequately, but is also flexible enough to handle these deviations.

Applying the gamma-Gompertz-Makeham model (ΓGM) to reconstruct adult age mortality is justified for at least three reasons (for more details of the models see Methods). First, it captures both excess mortality at young-adult ages and the deceleration of death rates at older ages [6, 7]. Second, the model is able to capture both an infinitely increasing risk of death and an S-shaped pattern that reflects an eventual mortality plateau, i.e., the asymptotic convergence of mortality rates at the oldest-old ages [8–12]. Third, at the oldest ages it is qualitatively similar to the Kannisto model [7] applied by the largest high-quality mortality databases [3, 13, 14].

Using the ΓGM model instead of the Kannisto model has several important advantages. First, the ΓGM has one extra parameter, *γ*, that gives information about the magnitude of unobserved heterogeneity (frailty) in the population [15]. In addition, when *γ* = 0, the model is able to capture a pure Gompertz increase of death rates whereas the Kannisto model always assumes deceleration at older ages. Moreover, for *γ* > 0, the ΓGM asymptote of death rates, equal to $\frac{b}{\gamma }$, can be any positive number while in the Kannisto framework it is restricted to 1. This could play a crucial role in comparing survival probabilities for both sexes or various populations at the oldest-old ages.

The second extra parameter, the Makeham term [16], captures extrinsic mortality at younger ages and ensures that the model is less sensitive to the starting age of analysis [17]. To avoid the non-negligible effect of extrinsic mortality, the Kannisto model is usually fitted starting from at least age 80 onwards [15, 18–20], whereas the ΓGM can be fitted over wider age ranges.

Fitting a ΓGM model is not only adequate for the right tail of the mortality distribution, but it also provides a straightforward expansion of abridged (grouped) life-table values to (non-)integer ages. This aids life-expectancy reconstruction for historical populations with scarce data.

Here, we aim to to quantify the difference between ΓGM life expectancy values and those published in the largest public high-quality mortality databases, as well as draw attention to certain populations for which life expectancy values should be reconsidered.

## Methods

The most commonly used procedure for life tables in the open-ended age group is to assume that the average number of person-years lived by the individuals dying in this age group equals the reciprocal of the death rate in this age group [1]. Suppose that *x*_*c*_ is the censoring age for a life table and above this age age-specific information is aggregated. Using standard life-table algebra with a constant-hazard assumption, remaining life expectancy for this open-ended age group is given by

$$e_{x_{c}}=\frac{1}{m_{x_{c}}},$$

where $m_{x_{c}}$ is the death rate corresponding to this age group.

Closing a life table according to another assumption leads to a completely new set of remaining life expectancy values at all ages as each assumption imposes a particular structure of mortality in the last age group. If the assumption does not reflect the real age pattern of mortality in this group, then mortality measures based on life tables can be distorted. To avoid the latter, it is necessary to treat right-censoring adequately—in a standard survival analysis setting by applying a parametric model that describes well the mortality mechanism. The abundance of historical life tables and the large size of human populations result in regular mortality patterns that can be well described by a Gompertz model adjusted for deviations at its ends (ΓGM).

We assume that death counts at age *x*, *D*(*x*) are Poisson-distributed [21]: *D*(*x*) ∼ *Poisson*(*E*(*x*)*μ*(*x*)), where *E*(*x*) denotes the corresponding exposure at age *x* and *μ*(*x*) is the risk of death, or hazard, at this age. The hazard function for the gamma-Gompertz-Makeham model at age *x* is given by the following expression:

$$\mu \left(x\right)=\frac{ae^{bx}}{1+\frac{a\gamma }{b}\left(e^{bx}-1\right)}+c.$$

Parameter *a* denotes the level of senescent mortality at the starting age of analysis, *b* is the rate of individual aging, *c* is an age-independent external risk of death, and *γ* equals the squared coefficient of variation of the distribution of unobserved heterogeneity [22].

We estimate the parameters of the hazard by maximizing a Poisson log-likelihood in the form

$$\text{ln}L=\sum_{x}[D(x)\text{ln}\mu (x)-E(x)\mu (x)].$$

In case age-specific death counts and exposures are unknown and only death rates are available, we estimate model parameters by applying non-linear least squares.

Optimization was carried out by applying differential evolution [23] using the DEoptim R-package [24]. Applying differential evolution minimizes the risk of arriving at a local maximum of the likelihood.

For life expectancy at birth, we fit the ΓGM model from age 30 onwards and take survival probabilities directly from the life tables at preceding ages.

The Kannisto model [7] applied in the HMD, the WPP and the WHO databases is characterized by the following hazard function:

$$\mu \left(x\right)=\frac{ae^{b(x-x_{0})}}{1+ae^{b(x-x_{0})}}.$$

Parameter *a* indicates the level of senescent mortality at the starting age of analysis, *b* is the rate of individual aging and *x*_0_ is the starting age of analysis. Method protocols of these databases provide further details on the subtle differences each database implemented for its own estimation procedure [18–20].

## Results

**Fig 1:**
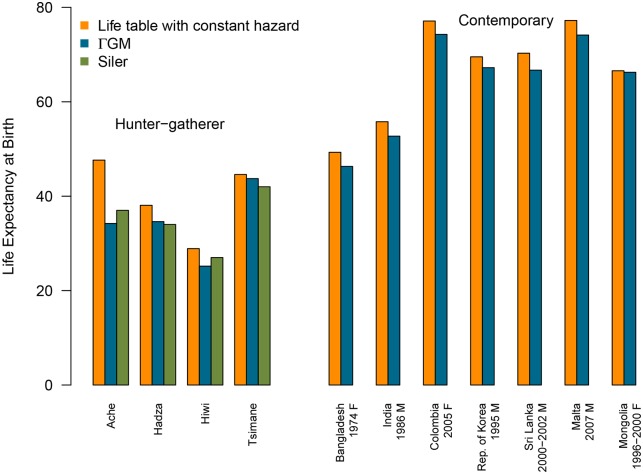
Life expectancy at birth for hunter-gatherer and contemporary populations. ΓGM was fitted from age 30 onwards. For hunter-gatherers life expectancies based on the Siler model estimated by [29] is also given.

For four hunter-gatherer populations presented in Fig 1 (data source: [25–28]), conventional life-table calculation overestimates the average length of life by 5.3 years (with a standard deviation of *σ* = 5.52 years). ΓGM estimates are closer to the ones of a Siler model (applied by the authors in [29]) that also assumes exponentially increasing adult mortality [30]. Note that mortality deceleration is not captured by the Siler model, which might be adequate for hunter-gatherer populations, but not for contemporary populations. This is the reason why we do not compare ΓGM and Siler life expectancies for human populations.

The Human Life-table Database [31] contains life tables of varying quality for national, subnational or ethnical subpopulations. For populations in Fig 1 the open-ended age group starts at age 70, 80 or 85, and the proportion of censored individuals reaches from 14.15% (Republic of Korea) up to 56.10% (Colombia). The difference in life expectancy at birth between the life-table values and the ΓGM estimates varies randomly (for details, see [2], p.6, Fig 5). On average ΓGM estimates 2.6 years lower life expectancies (*σ* = 1.074): from only 0.32 years of difference in the average lifespan of Mongolian females with 53.23% of censored population to 3.59 years for Sri Lanka males with only 15.55% censoring. Correcting life-expectancy values is crucial for such populations.

**Fig 2:**
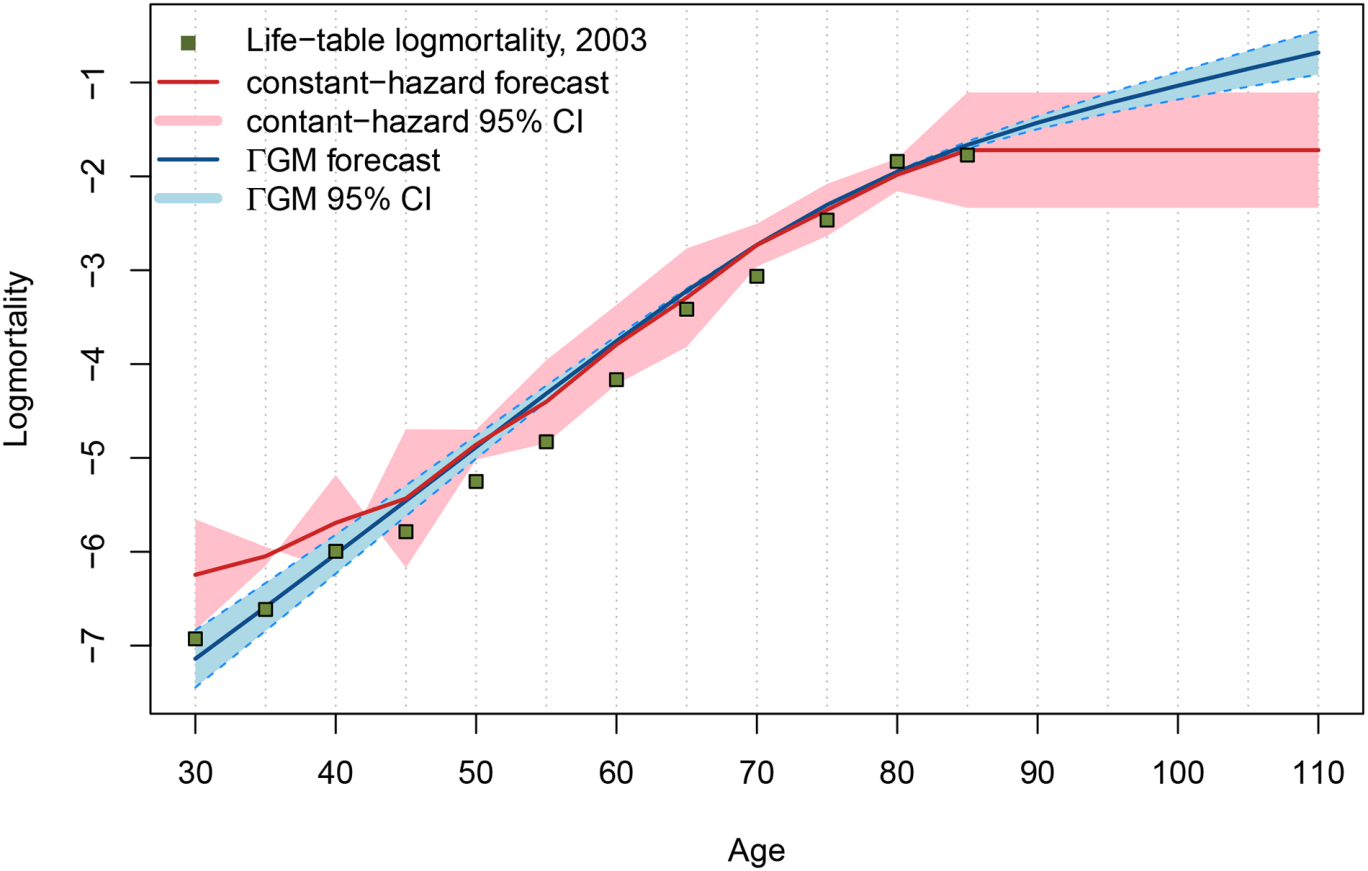
Lee Carter forecast for historical Bangladesh female data. Life-table data for year 2003 is designated by green squares. Forecasts based on the constant-hazard assumption and the ΓGM model are denoted by the red and blue curves, corresponding 95% confidence intervals with red and blue shaded areas, respectively.

Fig 2 compares Lee-Carter forecasts [32] for the year 2003 based on mortality data for Bangladeshi females in the period 1984-1994 [31]. Life-table life expectancy for females equals 44.95 years in 2003. Assuming a constant hazard at the oldest ages, the forecast predicts 42.39 years, whereas the ΓGM model results in 42.53 years for 2003. Albeit neither of the forecasts captured the mortality reduction at adult ages that occurred in the forecast period, the difference of age-specific rates from the actual life-table values and the underestimation of life expectancy are smaller for the ΓGM model. Comparing the predicted mortality rates and the actual life-table values in Fig 2 shows that point estimates favor the ΓGM forecast in this case. However, the wider confidence bounds of the constant-hazard forecast tend to contain more of the actual life-table values at adult ages and neither of them captured the mortality reduction at adult ages that happened in the forecast period.

Forecasting life expectancy for the year 2010 based on Japanese female data from the period 1947-2000 with artificial censoring at ages 80,85 and 90 and a constant hazard results in 52.27, 49.85 and 49.15 years, respectively, whereas the forecast based on ΓGM estimates is 49.44 years. Hence, using the ΓGM model can be considered more robust to the influence of insufficient data [2].

Estimating life expectancy within the ΓGM and the Kannisto framework leads to almost identical results (more details in [2]). The combination of a piecewise cubic Hermite interpolating polynomial, Gompertz and Kannisto models applied by the UN (exact details of this estimation procedure can be found in the methodological notes prepared by the UN [18]) in [3] for different parts of the adult-mortality curve can be substituted by estimating just the ΓGM model for adult ages, starting from ages succeeding the accident-related mortality hump. As a result, for all life tables in the period 1950-2015, ΓGM estimates are higher by only 0.422 years (*σ* = 0.503) on average than published life-table life expectancies.

Life tables in the Human Mortality Database [13] end at age 110 and only a very few individuals are censored. Raw death-count and exposure data are available, but all published life tables are Kannisto-smoothed, which implies that the resulting life expectancies are almost identical to the ΓGM estimates for these populations. The World Health Organization constructs life tables with the Kannisto model, and after applying graduation techniques, mortality rates are harmonised with those in [13] and [3]. As a result, ΓGM estimates are very close to the published life expectancies there, too.

Eurostat constructs life tables using a harmonized framework based on data provided by national statistical offices [33]. In spite of the fact that the open-ended age interval starts at age 85 for the currently published life tables that use standard life-table algebra, life-expectancy values are within close range of those published in the HMD. ΓGM estimates based on adult ages (30+) excluding the open-ended age group lead to similar life expectancies. This suggests that death rates for the last age group are chosen to match the remaining life expectancy value for this group based on a parametric model estimated in advance on data at ages above 85 included (see discussion and Fig 5 in [2]). Differences from the ΓGM estimates could be due to the different (possibly Kannisto) model fitting on different age ranges and age-specific data at older ages that include more variation.

Given the proximity of ΓGM-based life expectancy values to the ones in the HMD, UN, WHO, and Eurostat databases, each of which applies a set of models to arrive at the officially published mortality data version, we suggest fitting just a ΓGM at adult ages.

## Conclusion

Reconstructing adult human mortality within the ΓGM framework is essential for life tables in which a substantial proportion of the population are censored. Life tables that do not address censoring appropriately, distort life-expectancy values and other dispersion measures of mortality based on them, e.g. life disparity [34], Keyfitz’s entropy [35], the Gini coefficient [36], and the coefficient of variation (see e.g. [37]). In this article, we find such evidence for human (e.g. Bangladesh, females, 1974; Malta, males, 2007, etc.) and hunter-gatherer populations.

The largest high-quality mortality databases available—[3, 13, 14]—cope successfully with the aggregation of data at the oldest ages. Nevertheless, they might consider applying a single ΓGM model starting from age 25-30 approximately, instead of a composition of separate techniques and estimates for various parts of the adult mortality curve. Fitting a Kannisto and a ΓGM model is statistically identical, but estimated parameters of the latter can shed light on the shift of mortality patterns to older ages, the rates of individual and population aging, the amount of population heterogeneity, as well as the onset and magnitude of the mortality plateau. Given these beneficial features of the ΓGM model, we advocate analyzing adult human mortality within this framework.

## Supporting information

S1 Text Supplementary material on the presented data. (PDF)
