# Supplementary material for: Adequate life-expectancy reconstruction for adult human mortality data
Source: PLoS One. 2018 Jun 4;13(6):e0198485. doi: 10.1371/journal.pone.0198485 (PMC5986123; doi:10.1371/journal.pone.0198485)
Supplement: S1 Text — (PDF) [file pone.0198485.s001.pdf]

Supporting Information

The hunter-gatherer data are published in [1] for the Ache, in [2] for the Hadza, in [3] for the Hiwi and in [4] for the Tsimane populations.

Table 1. Life expectancy at birth for hunter-gatherer populations in Fig 1.

Population	Sex	Life-table e_0	ΓGM e_0	Siler e_0
Ache	Female	47.64	34.22	37
Hadza	Female	38.07	34.61	34
Hiwi	Female	28.89	25.18	27
Tsimane	Male	44.59	43.72	42

The contemporary human population data analyzed in the manuscript are freely available in the Human Mortality Database [5], the Human Life-table Database [6], the World Population Prospects [7], and the World Health Organization Global Health Observatory [8] and the Database maintained by Eurostat [9].

Table 2. Life expectancy for populations from [6] in Fig 1.

Population	Period	Sex	Censoring Age	Life-table e_0	ΓGM e_0
Bangladesh	1974	Female	70	49.30	46.31
India	1986	Male	70	55.78	52.72
Colombia	2005	Female	80	77.11	74.28
Republic of Korea	1995	Male	85	69.53	67.23
Sri Lanka	2000-2002	Male	85	70.29	66.70
Malta	2007	Male	85	77.23	74.14
Mongolia	1996-2000	Female	70	66.57	66.25

References

1. Hill KR, Hurtado AM. Ache Life History: The Ecology and Demography of a Foraging People. New York: Aldine de Gruyter; 1996.

2. Blurton Jones NG, Hawkes K, O'Connell JF. Antiquity of postreproductive life: Are there modern impacts on hunter-gatherer postreproductive life spans? American Journal of Human Biology. 2002;14(2):184–205. doi:10.1002/ajhb.10038.

3. Hill K, Hurtado AM, Walker RS. High adult mortality among Hiwi hunter-gatherers: Implications for human evolution. Journal of Human Evolution. 2007;52(4):443–454. doi:10.1016/j.jhevol.2006.11.003.

4. Gurven M, Kaplan H, Supa AZ. Mortality experience of Tsimane Amerindians of Bolivia: regional variation and temporal trends. American Journal of Human Biology. 2007;19(3):376–398. doi:10.1002/ajhb.20600.

5. HMD. The Human Mortality Database; 2017. http://www.mortality.org/.

6. HLD. The Human Life-Table Database; 2017. http://www.lifetable.de/.

7. WPP. The 2015 Revision of World Population Prospects; 2015. Available from: http://esa.un.org/unpd/wpp/Download/Standard/Mortality/.

8. WHO. World Health Organization Global Health Observatory Data Repository; 2017. http://www.who.int/gho/mortality_burden_disease/life_tables/life_tables/en/.

9. Eurostat. Eurostat Database and metadata information; 2018. http://ec.europa.eu/eurostat/data/database, http://ec.europa.eu/eurostat/cache/metadata/Annexes/demo_mor_esms_an1.pdf, Accessed: 2018-01-11.
